# DIMPL: a bioinformatics pipeline for the discovery of structured noncoding RNA motifs in bacteria

**DOI:** 10.1093/bioinformatics/btab624

**Published:** 2021-09-15

**Authors:** Kenneth I Brewer, Glenn J Gaffield, Malavika Puri, Ronald R Breaker

**Affiliations:** Department of Molecular Biophysics and Biochemistry, Yale University, New Haven, CT 06520-8103, USA; Howard Hughes Medical Institute, Yale University, New Haven, CT 06520-8103, USA; Department of Molecular, Cellular and Developmental Biology, Yale University, New Haven, CT 06520-8103, USA; Department of Molecular Biophysics and Biochemistry, Yale University, New Haven, CT 06520-8103, USA; Howard Hughes Medical Institute, Yale University, New Haven, CT 06520-8103, USA; Department of Molecular, Cellular and Developmental Biology, Yale University, New Haven, CT 06520-8103, USA

## Abstract

**Summary:**

Recent efforts to identify novel bacterial structured noncoding RNA (ncRNA) motifs through searching long, GC-rich intergenic regions (IGRs) have revealed several new classes, including the recently validated HMP-PP riboswitch. The DIMPL (Discovery of Intergenic Motifs PipeLine) discovery pipeline described herein enables rapid extraction and selection of bacterial IGRs that are enriched for structured ncRNAs. Moreover, DIMPL automates the subsequent computational steps necessary for their functional identification.

**Availability and implementation:**

The DIMPL pipeline is freely available as a Docker image with an accompanying set of Jupyter notebooks. Full instructions for download and use are available at https://github.com/breakerlab/dimpl.

**Supplementary information:**

Supplementary data are available at *Bioinformatics* online.

## 1 Introduction

Discovery and validation of the over 45 known classes of metabolite- or elemental ion-binding riboswitches (McCown *et al.*, 2017) have relied extensively on large-scale computational approaches based on comparative sequence analysis (Weinberg *et al.*, 2007, 2010, 2017). However, these large-scale approaches may struggle to identify new classes of riboswitches, which are predicted to exist by the thousands but are likely much rarer than known classes (Breaker, 2011; McCown *et al.*, 2017). Genome-level filtering of bacterial intergenic regions (IGRs) by nucleic acid composition and length (Brewer *et al.*, 2021; Meyer *et al.*, 2009; Stav *et al.*, 2019) was developed to address the challenges of discovering these rarer riboswitch classes. This approach has already enabled the discovery and validation of the SAM-V (Meyer *et al.*, 2009; Poiata *et al.*, 2009), HMP-PP (Atilho *et al.*, 2019) and NAD-II (Panchapakesan et al. 2021) riboswitch classes and the discovery of dozens of new intergenic motif candidates in the first genomes analyzed. However, until now this approach has required time-consuming manual analysis using several bioinformatic tools and lacked well-defined techniques to define genomic regions for further analysis that are enriched for noncoding RNAs (ncRNAs). 

In this article, we introduce DIMPL (Discovery of Intergenic Motifs PipeLine), a bioinformatics pipeline which automates the process of total genome analysis by extracting IGRs, filtering them by length and nucleic acid composition, and collecting the data necessary to identify candidate motifs and assign their possible functions. DIMPL also provides reproducible techniques for identifying genomic regions enriched for ncRNA through support vector machine (SVM) classifiers. Although our primary objective in creating DIMPL was to accelerate the discovery of novel riboswitch classes, it can also be used to identify a wide-range of other intergenic nucleic acid and protein motifs such as upstream open reading frames, short open reading frames, ribosomal protein leader sequences, selfish genetic elements and other structured RNA motifs of unknown function.

## 2 Results

### 2.1 Pipeline overview

**Fig. 1.**
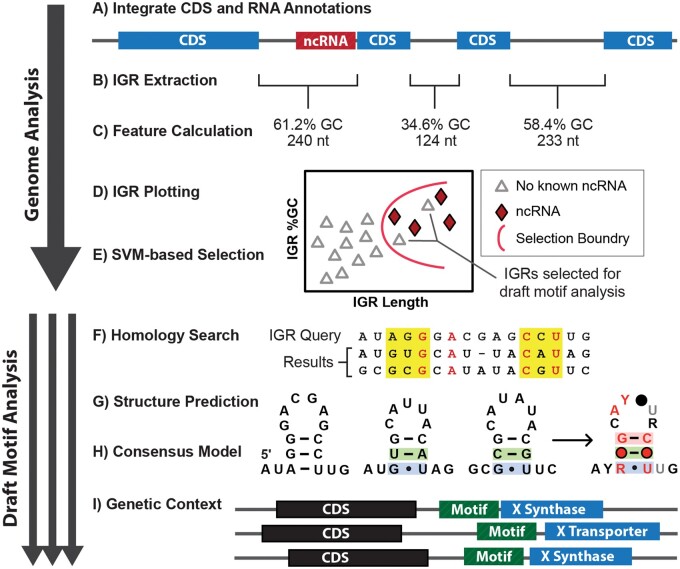
Overview of DIMPL. Process are divided into the two stages: genome analysis (**A**–**E**) and draft motif analysis (**F**–**I**). Annotations for sections D (Stav *et al.*, 2019) and H (Rivas *et al.*, 2017; Weinberg *et al.*, 2011) have been reported previously

The DIMPL computational pipeline consists of two primary stages: (1) genome analysis and (2) draft motif analysis. For the genome analysis stage of DIMPL, the user begins by entering the Uniprot ID for a microbial genome for which there are Rfam annotations. DIMPL proceeds to automatically request the latest genomic sequence and protein annotations (Fig. 1A) accessible via NCBI Entrez (Agarwala *et al.*, 2016) and the corresponding RNA family annotations provided by the Rfam MySQL Database (Kalvari *et al.*, 2018). All IGRs located between protein-coding open reading frames are then extracted and labeled (Fig. 1B and C) with their percentage of G and C nucleotides relative to the total nucleotides in the IGR (%GC content), length and the presence of any known ncRNA motifs. DIMPL then generates an interactive graph (Fig. 1D) showing the IGRs plotted by their %GC content and length with labels for IGRs with known RNA families. This genome plot can help evaluate the suitability of the selected genome for analysis using the GC-IGR search approach. Ideal genomes will have strong separation between the cluster of IGRs containing known ncRNAs and those the bulk of IGRs with no known annotation.

In the next step, the tool uses a SVM classifier (Fig. 1E) to identify IGRs with no annotated ncRNAs that have similar GC content and length to other IGRs with known structured ncRNAs. DIMPL then performs a BLASTX search (Camacho *et al.*, 2009) on the selected IGRs to ensure they do not contain unannotated protein coding regions. Any unannotated protein coding regions discovered in the search are removed from the selected IGRs, which are discarded in their entirety if the remaining IGR no longer meets the length and %GC content requirements for the selection.

The draft motif analysis portion of DIMPL is performed in parallel on all IGRs that have met the selection criteria. The process begins by using Infernal 1.1.3 (Nawrocki and Eddy, 2013) to search each selected IGR’s sequence (Fig. 1F) against a database of all microbial IGRs derived from NCBI’s RefSeq (O’Leary *et al.*, 2016). The collection of homologous sequences from a single IGR search forms the ‘draft motif’ that is further analyzed in several steps. First, representatives with identical nucleotide sequences are removed. Next, the draft motifs are analyzed via CMfinder 0.4.18 (Yao *et al.*, 2006) to look for possible RNA secondary structure features (Fig. 1G). All realigned motifs generated by CMfinder are evaluated for evidence of statistical significance for predicted nucleotide covariations. Subsequently, the consensus sequence and structural model for each motif is generated (Fig. 1H) using R-scape 1.4.0 (Rivas *et al.*, 2017), which integrates the RNA drawing algorithm R2R (Weinberg *et al.*, 2011). Draft motifs are also checked for the presence of coding regions using RNAcode (Washietl *et al.*, 2011). Finally, for each draft motif, DIMPL uses GenomeView (Spies *et al.*, 2018) to visualize the genetic contexts (Fig. 1I) of the motif’s representatives to aid in determining a possible function for the candidate RNA motif. A draft motif’s most strongly supported alignment can then be analyzed by one or more additional cycles of Infernal homology searches, which take advantage of the proposed secondary structure to expand the number of representatives found.

### 2.2 Details on SVM enrichment

The SVM enrichment of IGRs in DIMPL uses a radial basis-function (RBF) kernel and is implemented with scikit-learn (Pedregosa *et al.*, 2011). The SVM classifier is trained *de novo* for each genome analyzed using the IGR %GC content and nucleotide length as the features, the presence/absence of a structured RNA as the class labels and a set of hyperparameters that have been weighted to select a contiguous region of a genome’s %GC versus length plot. The primary purpose of the SVM classifier is to perform an enrichment of IGRs that reduces the number subjected to the more computationally intensive steps in the pipeline. Applying the SVM-RBF algorithm allows DIMPL to accomplish this goal in a systematic and reproducible manner.

### 2.3 Usage

The DIMPL pipeline is built primarily in Python and is distributed as a Docker image (Merkel, 2014) with all the necessary tools already installed. Along with the Docker image, DIMPL includes a set of detailed Jupyter notebooks that walk users through the steps of the pipeline, display interactive graphs and assemble results from analysis tools. For computationally intensive steps such as BLAST, Infernal and CMfinder that are typically performed on a high-performance computing cluster, DIMPL exports compressed tar files containing the necessary bash scripts and data files that can be configured for a custom compute environment. Detailed instructions are included in the Supplementary Information of this article. Sample datasets, preprocessed search database files, and the source code are available at www.github.com/breakerlab/dimpl. 

## 3 Conclusion

DIMPL provides an integrated collection of tools to streamline the process of identifying novel structured ncRNA motifs, including new riboswitch candidates, on a genome-wide scale. It relies on established methods of enriching bacterial IGRs for ncRNA motif discovery (Stav *et al.*, 2019) and quickly assembles the combination of structural and genetic context information that are key to identifying the function of the newly discovered motifs. This pipeline should permit the rapid analysis of each new bacterial genome for novel and rare ncRNA classes, which will aid in the discovery of novel classes of riboswitches and ribozymes.

## Supplementary Material

btab624_Supplementary_Data
