# Supplementary material for: DIMPL: a bioinformatics pipeline for the discovery of structured noncoding RNA motifs in bacteria
Source: Bioinformatics. 2021 Sep 15;38(2):533–5. doi: 10.1093/bioinformatics/btab624 (PMC8723152; doi:10.1093/bioinformatics/btab624)
Supplement: btab624_Supplementary_Data [file btab624_supplementary_data.docx]

***Supplementary Information***

**DIMPL: A bioinformatics pipeline for the discovery of structured noncoding RNA motifs in bacteria.**

**Kenneth I. Brewer^1^, Glenn J. Gaffield^2^, Malavika Puri^3^ and Ronald R. Breaker ^1,2,3,*^**

^1^Department of Molecular Biophysics and Biochemistry, ^2^Howard Hughes Medical Institute, ^3^Department of Molecular, Cellular and Developmental Biology, Yale University, P.O. Box 208103, New Haven, CT 06520-8103, USA.

*To whom correspondence should be addressed.

Contact: Ronald R. Breaker

Email: [ronald.breaker@yale.edu](mailto:ronald.breaker@yale.edu)

Phone: 203 432-9389

# Usage Manual

### Workstation Setup and Installation

Prerequisites:

- Install the Docker program (<https://www.docker.com/products/docker-desktop>)
- Ensure Python version 3 is installed on your machine.
- Ensure you have an NCBI account and API Key.

Setup

- Download the 5.7 GB Docker image for dimpl using the command:

docker pull breakerlab/dimpl

- Configure the settings of docker to allow the virtual machine to access local files:
  - Docker settings → Resources → File sharing → Select the local drives you want to be available to the docker image.
- Download the archive of DIMPL source from github.com/breakerlab/dimpl and extract to folder accessible to docker.

wget https://github.com/BreakerLab/dimpl/archive/v1.0.0.tar.gz

tar xzvf v1.0.0.tar.gz

- There will now be a dimpl folder in this repository.

cd dimpl-1.0.0; ls

Starting DIMPL

- To start dimpl, run the start.sh script:

./start.sh

- - For first time DIMPL users, a prompt will appear asking for NCBI account information (email address and API key). This information is found in your NCBI account, in account settings. If you do not already have an API key created, you can create one on this page.
- A URL will be output to access the jupyter notebook: (e.g. localhost:8888/lab… etc.). Copy and paste this url into your web browser.

Using Jupyter Notebooks

By default, DIMPL launches a JupyterLab interface that includes an integrated file browser, terminal, and access to the three computational notebooks that walk users through the DIMPL analysis pipeline. The “work” directory contains all the folders accessible to DIMPL from your local machine. The three main pipeline notebooks can be found in the “notebooks” directory. Each notebook is intended to be run one after the other, running each python code block in order and following the built-in instructions about which variables should be modified. An overview of each of the three computational notebooks is included here with more detailed instructions built into each notebook itself.

### Compute Station Setup

Compute Station Prerequisites

The following programs need to be installed and available on each node of the cluster that you plan to use:

- Slurm v20.02+ (Yoo *et al.*, 2003) Job scheduler
- Dead Simple Queue v1.05 (Evans and Bjornson, 2021) Job array tool
- Infernal v1.1.4 (Nawrocki and Eddy, 2013) Covariance model search tool
- BLAST+ 2.11.0+ (Camacho *et al.*, 2009) Sequence similarity search tool
- CMfinder v0.4.1.18 (Yao *et al.*, 2006) RNA motif predictor

Data to be processed (tar files) needs be placed in a location that is shared by all compute nodes.

Memory required per node:

- Blast step 70GB
- Infernal step 32GB

Downloading Necessary Files

- Download the IGR search database (filename: s50.igr.fasta) from the link on the DIMPL Github to your cluster using Globus FTP (Allcock *et al.*, 2005). This file is 91GB.
- Ensure the availability of the BLAST “nr” database on your computational cluster. This database is currently 547GB.

Modifying Configuration File

The cluster configuration file found at dimpl/src/shell/cluster.conf is used to modify your PATH environmental variables on your cluster such that all of the utilities mentioned under “Prerequisites” can be run. It also defines a few variables that define your cluster partition (queue) and database locations. Although this file is used on the compute cluster, it should be modified within the workstation dimpl installation as the config file is packaged with each tarfile of computational instructions assembled by DIMPL.

### Notebook 1: Genome IGR Selection

Introduction

The purpose of DIMPL Notebook 1 is to walk the user through the steps of selecting a bacterial genome, extracting and graphing the intergenic regions, and refining the Support Vector Machine (SVM) selection parameters used to enrich the intergenic regions for structured non-coding RNAs.

Step 1: Review bacterial/archaeal genomes annotated by Rfam

Run the first code block to make all the necessary python imports, then run the second code block to create a connection to the Rfam mySQL database (Kalvari *et al.*, 2018) and request a list of annotated genomes from the Bacteria and Archaea kingdoms. This code block demonstrates some of the various filtering capability for genome selection that can be accomplished using the built-in functionality of the SQLAlchemy classes used to query Rfam’s database. By default, this code block will output a table with the information for the first 10 genomes, but this can be modified by changing the indices in the code block. Take note of the Uniprot ID (UPID) of the genome you wish to analyze and enter it in the code block below.

Step 2: Extract and graph IGRs

For this example, we will select the genome of *Campylobacter jejuni* (UPID: UP000000799). Replace the upid variable in code block 3 with the value for this genome and then run the code block. This code block will automatically identify the correct genome from NCBI, download the necessary files, extract the genome’s IGRs, annotate the IGRs using data available from Rfam and then generate the plot of the IGRs as shown in **Figure 1.**  This particular genome shows some of the ideal characteristics for genomes that are relatively easy to analyze using the GC-IGR analysis approach. There is a relatively low overall GC content, which results in the IGRs containing known structured ncRNAs having good separation on the plot from other IGRs.

Step 3: SVM selection of IGRs enriched for ncRNAs.

Running the next code block will create a similar IGR plot for the selected genome, but will use a radial basis function (RBF) kernel with a support vector machine classifier to create a selection of intergenic regions that are enriched for structured noncoding RNAs (**Figure 2**). Above the plot are sliders for adjusting three hyperparameters that go into defining the IGR selection: 1) *class_weight*, 2) *gamma_exp* and 3) *c_exp*. Researchers are seeking a greater understanding of the role of these hyperparameters in SVM classifiers are advised to consult the documentation of Scikit-learn (Pedregosa *et al.*, 2011). In short, a proper selection of hyperparameters is necessary to prevent overfitting while training an SVM classifier. DIMPL performs the SVM classifier training on a genome-by-genome basis, however, we have found that the default hyperparameters shown in this code block generate reasonable enrichment for a wide-range of genomes. Generally, we advise users to rely on these default parameters unless analyzing a genome with truly unusual IGR characteristics.

Step 4: Finalize IGR Selection and export blast tarfile

Running the final two code blocks in Notebook 1 will re-create the selection and write the data into a comma separated value file called ‘annotated_igrs.csv’. DIMPL will then generate appropriate fasta files and scripts necessary to perform a BLAST search on the selected IGRs to look for unannotated ORFs. These fasta files and scripts are packaged into a tarfile that is placed in DIMPL’s data/export folder. Transfer this tarfile to your computational cluster, unzip the tarfile and execute the script “./blast_run.sh” to start the series of blast searches on the selected IGRs. Progress of all the different tasks can be monitored in slurm using the “squeue -u username” command. Once all computational tasks are completed, run the “./make_tar.sh” command to package the results folder for the next processing step.

### Notebook 2: BLAST data processing

Introduction

The purpose of DIMPL’s notebook 2 is to process the BLAST results generated using Notebook 1 to identify IGRs that contain unannotated known ORFs and should either be discarded or trimmed. After importing the Blast results, DIMPL determines how IGRs need to be trimmed by calculating a customizable “orf_score” at each nucleotide position in the IGR. This orf_score is a sum of quantity and quality of BLAST hits that overlap that position. After removing all portions of an IGR that meet the orf_score threshold, the notebook checks if any of the remaining IGR or IGR fragment still falls within the original IGR selection boundary. Finally, the notebook builds a new set of scripts required to start the Infernal searches on each remaining IGR and assembles those into a tarfile.

Step 1: Import BLAST search files

Transfer the Blast results tarfile generated at the end of Notebook 1’s instructions and place it in the data/import folder. After running code block 1, change the variables in code block 2. The import_tar_name should be changed to the name of the tarfile placed in data/import. For the *Campylobacter jejuni* example described above the assembly_acc variable should be set to “GCA_000009085.1” and selection_name may be left unchanged. Code blocks 2, 3, and 4 should then be run to extract the BLAST results from the tarfile, and rebuild the original selection used for a genome.

Step 2: Process BLAST results

Run code block 5 to analyze the blast results for each IGR and calculate an orf_score at each nucleotide position as depicted in **Figure 3**. The parameters used for trimming IGR portions can be customized by changing the arguments of the process_blast() function in this code block. For poorly annotated genomes where the vast majority of IGRs contain unannotated ORFs, there may be very few of the resulting trimmed IGRs that meet the original GC/length selection criteria as defined by the SVM decision surface. In these cases, selection criteria may be relaxed by decreasing the svm_decision_cutoff from 0 to a slight negative value such as -0.3. DIMPL will visualize the impact of the slightly relaxed selection criteria by replotting the selected genome with the revised criteria. Any processed IGRs that fall in the highlighted area according to their recalculated length and GC content will be included in the subsequent steps.

Step 3: Build data and script tarfiles

Run code block 6 to assemble all of the processed IGRs into a new tarfile along with the necessary scripts to perform an Infernal(Nawrocki and Eddy, 2013) search on each IGR. You may customize the step_dir variable if you anticipate performing multiple rounds of infernal searches on this genome each of which can be placed into an appropriately named subfolder. Note that this codeblock calls the build_infernal_commands() function with the no_secondary_structure parameter set to “True”. This parameter instructs Infernal to use its nhmmer fallback option to handle these alignment files which contain only one sequence each and no predicted secondary structure. After running code block 6, find the resulting tarfile in the data/export folder, transfer it to your high-performance computing cluster, unpack the tar archive, and then run the command ./infernal_step1_run.sh. After all the computational steps have completed, assemble the data using the ./make_tar.sh command and transfer it back to DIMPL for analysis in Notebook 3.

### Notebook 3: IGR Report

Introduction

The purpose of Notebook 3 is to display and assemble the results of the Infernal search for each IGR from your selected genome. In addition to showing a results table with the number of hits and their locations, this notebook will display a variety of possible structures for each motif candidate predicted using CMfinder (Yao *et al.*, 2006) and R-scape (Rivas *et al.*, 2016). It will also run an analysis using RNACode (Washietl *et al.*, 2011) to check if the collection of hits is predictive of a novel protein-coding region. Finally, DIMPL generates genome context images that show the predominant gene associations upstream and downstream of each representative. The combination of structural information and genetic context information aids in the categorization of each motif candidate.

Step 1: Import infernal search results

Place the tarfile generated at the end of the instructions for Notebook 2 in the data/import folder. Run code block 1 and then change the variables in code block 2 to reflect the information for the genome being imported just as previously described for Notebook 2. Running code blocks 2 and 3 will generate a drop-down menu with an option for each IGR’s search results. Select the search results you are interested in viewing from this menu.

Step 2: View results table

Run code block 4 to generate an abbreviated results table that shows the number of unique representatives found for this motif along with the e-values and locations of these hits.

Step 3: View possible structures predicted by Rscape and CMFinder

Running code block 5 will generate a structure prediction using R-scape’s CaCoFold (Cascade variation/covariation Constrained Folding) algorithm(Rivas, 2020). The next code block will display the collection of possible submotifs predicted by CMFinder. The most well-supported structure may be one of these submotifs that might only be present in a subset of the homologous sequences that Infernal initially found. After viewing all of the possible structures, select the most promising structure and find the corresponding Stockholm alignment file in a new folder created with the structure data/motif_collections/{collection_name}/{step_name}/motifs in DIMPL’s folder architecture for further analysis.

Step 4: Perform RNAcode analysis to search for possible protein-coding regions

Run code block 7 to start an RNAcode analysis on this motif candidate to check if there are indications of novel protein-coding regions in this motif.

Step 5: View motif’s genome context

Running code block 7 will generate genome context images (**Figure 4**) for the motif. This allows a user to view the genes present upstream and downstream of each representative. A link to the NCBI genome browser of the genomic location is also provided which allows easy access to more detailed information about neighboring genes.

### Notebook 4: Motif Refinement

Introduction

The purpose of DIMPL’s notebook 4 is to perform follow-up searches on the initial motifs generated using Notebook 3.

Step 1: Collect selected RNA Alignments

The batch of stockholm files for the motifs selected for future analysis in Notebook 3 Step 3 should have been saved in the DIMPL folder architecture as follows:

data/motifs_collections/{collection_name}/{step_name}/motifs

A possible naming structure for these folders might be c_jejuni_motifs as the “collection_name” to represent a group of motifs originating from that organism’s genome. The “step_name” folder might be named infernal_step2 to represent the second round of homology search being performed on this group of motifs. Change the variables collection_name and step_name in code block 2 and run code blocks 1 to 3 to generate a tarfile of the sto files and scripts required for motif analysis.

Step 2: Transfer tarfile to HPC cluster, run, and collect results.

After running code block 3, find the resulting tarfile in the data/export folder, transfer it to your high-performance computing cluster, unpack the tar archive, and then run the command ./infernal_step2_run.sh. After all the computational steps have completed, assemble the data using the ./make_tar.sh command and transfer it back to DIMPL for continued analysis in Notebook 4.

Step 3: Import and view motif results in DIMPL.

Place the tarfile generated in the previous step in the data/import folder. Run code blocks 4-5 to generate a drop-down menu with an option for each IGR’s search results. Select the search results you are interested in viewing from this menu. Detailed instructions for each part of these motif refinement results are equivalent to those described for Notebook 3.

## Conclusion

DIMPL provides an integrated collection of tools to streamline the process of identifying novel structured ncRNA motifs, including new riboswitch candidates, on a genome-wide scale. It relies on established methods of enriching bacterial IGRs for ncRNA motif discovery and quickly assembles the combination of structural and genetic context information that are key to identifying the function of the newly discovered motifs. This pipeline should permit the rapid analysis of bacterial genomes for novel and rare ncRNA classes and can help accelerate the pace of riboswitch and ribozyme discovery.

### Figures


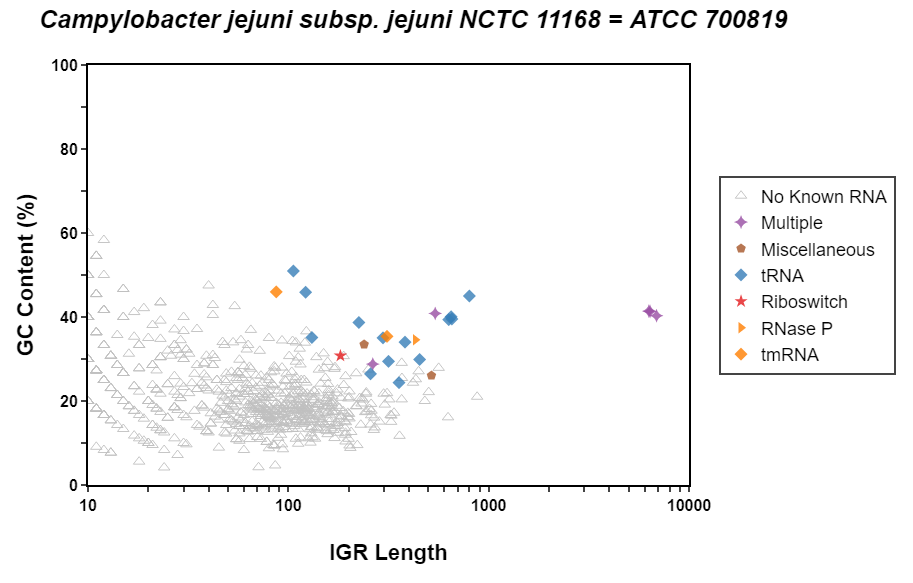


#### Figure 1: Plot of IGRs from the genome Campylobacter jejni generated by DIMPL.

Example of the genome plot generated by DIMPL When viewed in the DIMPL’s ingegrated Jupyter notebook this plot is interactive and specific IGRs annotations can be viewed by hovering over relevant points.


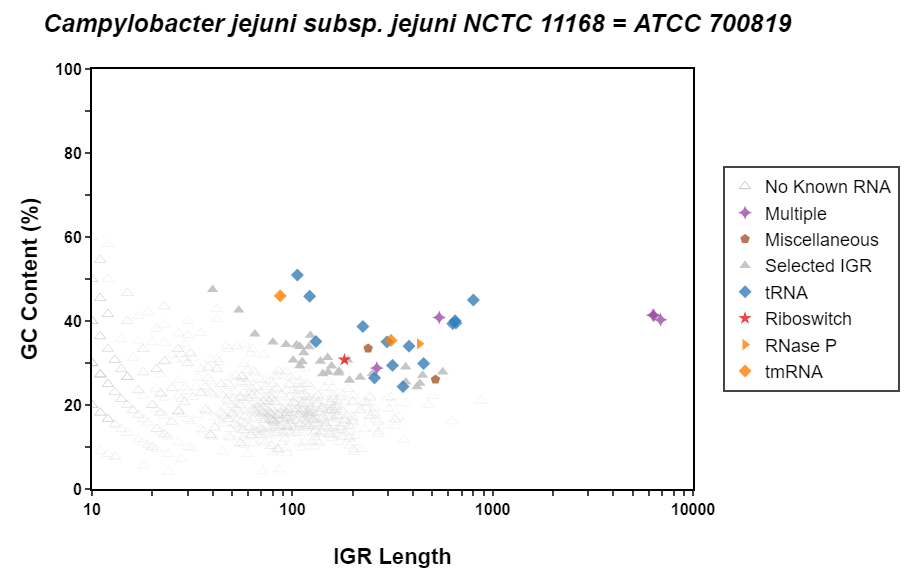


#### Figure 2: Plot of IGR selection from Campylobacter jejuni enriched by DIMPL

In contrast to **Figure 1**, this figure has IGRs with no annotated ncRNA highlighted if they are identified by DIMPL as having similar GC and length characteristics as those IGRs that do contain structured ncRNAs. This selection was generated using the default DIMPL hyperparameters of 0.50 for class_weight. 2.00 for c_exp and -2.00 for gamma_exp. In the Jupyter notebook that generates these selections, DIMPL also displays statistics about the selection including the number of IGRs with known ncRNAs included in the selection area (25 known ncRNAs out of 25 total ncRNAs or 100%), the number of unknown IGRs included in the selection regions (30 selected unknown IGRs out of 1008 total unknowns or 3.0%) and the fold enrichment for the selection (17.98 greater likelihood of finding ncRNA in selected region vs a random genomic IGR).


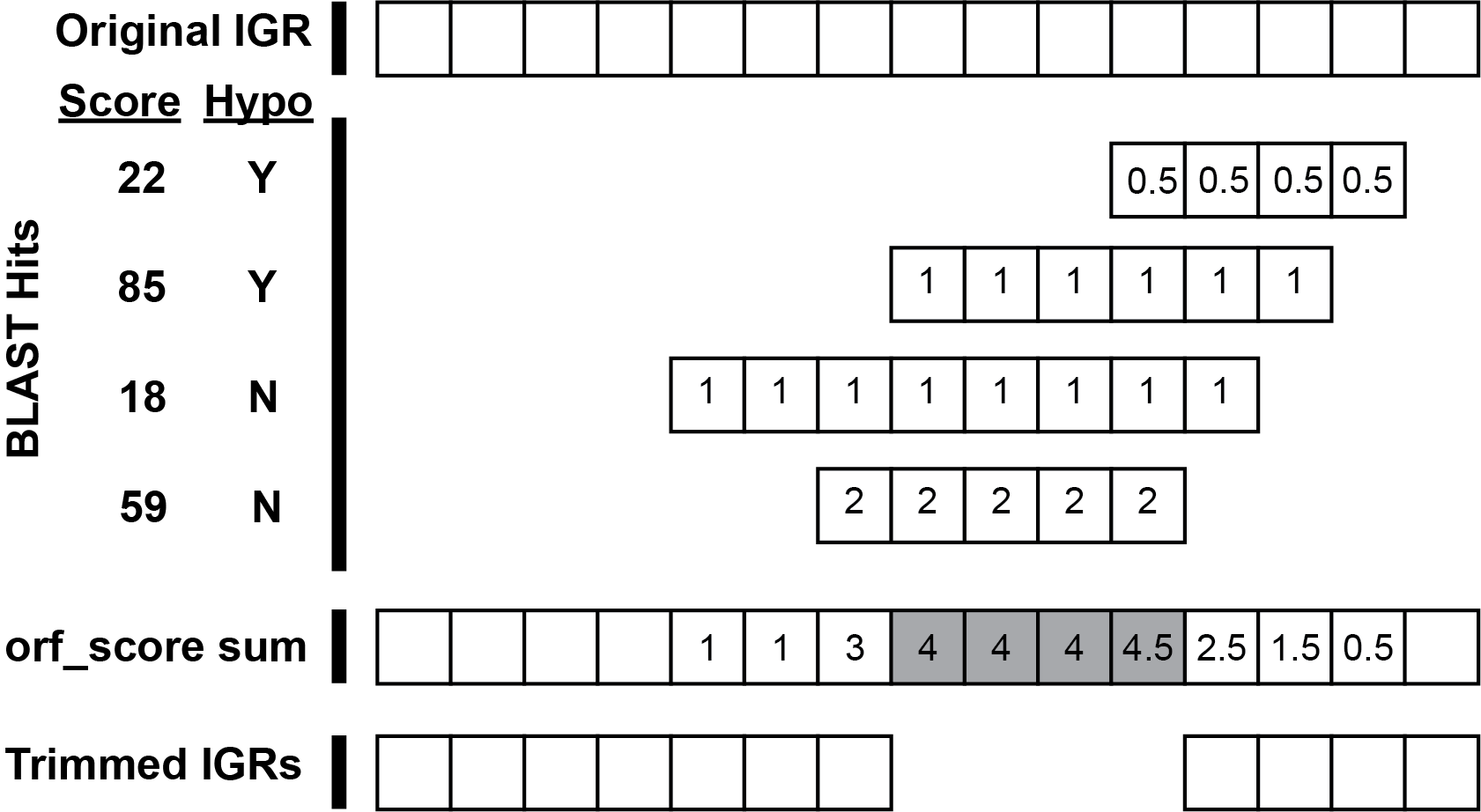


#### Figure 3: Graphical depiction of blast hit processing with default parameters

In this toy example, the BLAST search results for a 15 nt long IGR include four hits, each of which has data about the score, annotation, and region of overlap. Each hit is initially worth score_increment points (2) but that is multiplied by the poor_score_weight (0.5) if the quality of the hit is below the poor_blast_score (40) threshold. This is further multiplied by hypothetical_weight (0.5) if the hit includes variations of “hypothetical” in the annotation text. The sum of the resulting points at each nucleotide position is calculated and the central portion of the IGR which has an orf_score greater than orf_score_cutoff (4) is removed giving two new trimmed IGRs whose %GC content and length can be compared to the IGR selection parameters for possible inclusion.

**
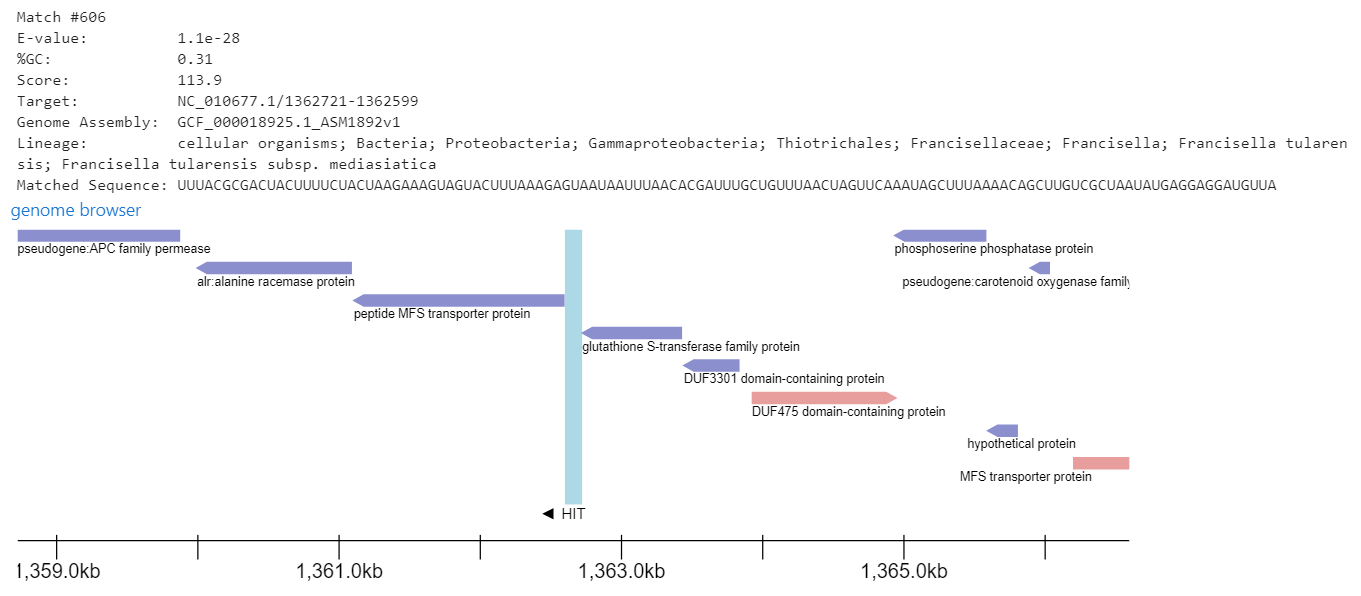
**

#### Figure 4: Sample hit from genetic context report generated by DIMPL

For each unique representative identified via an Infernal search DIMPL generates a genetic context report listing the e-value, genomic location, organism name and the sequence of the hit at that location. The motif is located blue-shaded region and is oriented with the 3’end towards the right. If the annotated gene names are uninformative additional detail is available by clicking the automatically generated link that will display the appropriate location in NCBI’s online genome browser(Agarwala *et al.*, 2016).

# References

Agarwala,R. *et al.* (2016) Database resources of the National Center for Biotechnology Information. *Nucleic Acids Res.*, **44**, D7–D19.

Allcock,W. *et al.* (2005) The Globus striped GridFTP framework and server. In, *SC’05: Proceedings of the 2005 ACM/IEEE conference on Supercomputing*. IEEE, p. 54.

Camacho,C. *et al.* (2009) BLAST+: architecture and applications. *BMC Bioinformatics*, **10**, 421.

Evans,B. and Bjornson,R. (2021) DeadSimpleQueue.

Kalvari,I. *et al.* (2018) Non-Coding RNA Analysis Using the Rfam Database. *Curr. Protoc. Bioinforma.*, **62**, 51.

Nawrocki,E.P. and Eddy,S.R. (2013) Infernal 1.1: 100-fold faster RNA homology searches. *Bioinformatics*, **29**, 2933–2935.

Pedregosa,F. *et al.* (2011) Scikit-learn: Machine learning in Python. *J. Mach. Learn. Res.*, **12**, 2825–2830.

Rivas,E. *et al.* (2016) A statistical test for conserved RNA structure shows lack of evidence for structure in lncRNAs. *Nat. Methods*, **14**, 45–48.

Rivas,E. (2020) RNA structure prediction using positive and negative evolutionary information. *PLOS Comput. Biol.*, **16**, e1008387.

Washietl,S. *et al.* (2011) RNAcode: Robust discrimination of coding and noncoding regions in comparative sequence data. *RNA*, **17**, 578–594.

Yao,Z. *et al.* (2006) CMfinder - A covariance model based RNA motif finding algorithm. *Bioinformatics*, **22**, 445–452.

Yoo,A.B. *et al.* (2003) Slurm: Simple linux utility for resource management. In, *Workshop on Job Scheduling Strategies for Parallel Processing*. Springer, pp. 44–60.
